# New perspectives of volemic resuscitation in polytrauma patients: a review

**DOI:** 10.1186/s41038-016-0029-9

**Published:** 2016-02-16

**Authors:** Ovidiu Horea Bedreag, Marius Papurica, Alexandru Florin Rogobete, Mirela Sarandan, Carmen Alina Cradigati, Corina Vernic, Corina Maria Dumbuleu, Radu Nartita, Dorel Sandesc

**Affiliations:** 1 Clinic of Anaesthesia and Intensive Care, Emergency County Hospital “Pius Brinzeu”, Bd. Iosif Bulbuca nr.10, Timisoara, Timis Romania; 2 Faculty of Medicine, “Victor Babes” University of Medicine and Pharmacy, Timisoara, Romania; 3 Clinic of Anaesthesia and Intensive Care “Casa Austria”, Emergency County Hospital “Pius Brinzeu”, Timisoara, Romania; 4 Faculty of Chemistry, Biology, Geography, West University of Timisoara, Timisoara, Romania

**Keywords:** Multiple trauma, Fluid resuscitation, Intensive care, Electrolyte imbalance

## Abstract

Nowadays, fluid resuscitation of multiple trauma patients is still a challenging therapy. Existing therapies for volume replacement in severe haemorrhagic shock can lead to adverse reactions that may be fatal for the patient. Patients presenting with multiple trauma often develop hemorrhagic shock, which triggers a series of metabolic, physiological and cellular dysfunction. These disorders combined, lead to complications that significantly decrease survival rate in this subset of patients. Volume and electrolyte resuscitation is challenging due to many factors that overlap. Poor management can lead to post-resuscitation systemic inflammation causing multiple organ failure and ultimately death. In literature, there is no exact formula for this purpose, and opinions are divided. This paper presents a review of modern techniques and current studies regarding the management of fluid resuscitation in trauma patients with hemorrhagic shock. According to the literature and from clinical experience, all aspects regarding post-resuscitation period need to be considered. Also, for every case in particular, emergency therapy management needs to be rigorously respected considering all physiological, biochemical and biological parameters.

## Background

Multiple trauma patient represents a challanging case in the intensive care unit (ICU). Severe systemic inflamatory syndrome trigger multiple biochemical and physiological dysfunctions resulting in a significant decrease in survival rate [1, 2]. Special attention is given to hemorrhagic shock [3, 4]. Volume replacement is often defining the clinical evolution of these patients. Impaired tissue oxygenation caused by insufficient perfusion has direct implications in anaerobic metabolism, inflammation and ultimately multiple organ dysfunctions syndrome (MODS) [5, 6]. Moreover, cellular and tissue hypoxia lead to abnormal celullar metabolism accompanied by redox imbalances. Mitochondrial dysfunction, release of pro-inflammatory molecules, cell adhesion factors, metabolites resulting from cell destruction are all responsible for synthesis of a significant amounts of free radicals (FR) [7] that can lead to the phenomenon called oxidative stress (OS) [8]. The best-known FR present in trauma patients are represented by hydroxyl radical, superoxide radical, hydrogen peroxide, nitroperoxide. The main sources of FR are represented by mitochondria, xanthine oxidase, nicotinamide adenine dinucleotide phosphate NAD(P)H oxidase, as well as a number of endothelial factors [9, 10].

Fluid therapy can be achieved by administering blood products and substitutes, crystalloid solutions (low molecular weight molecules able to cross the semi-permeable membranes) or colloidal solutions (high molecular weight molecules, unable to cross the semipermeable membrane) [11–13]. Most clinical studies are inconsistent regarding the use of solutions for volume replacement in hemorrhagic shock resuscitation. If some authors recommend crystalloid solutions, others recommend colloidal solutions, blood and blood product or mixed with different biologically active substances [14]. In this update has the propose, following a thorough literature review, to present current methods and implications of volume resuscitation in patients with multiple trauma.

## Review

### Pathophysiology of hemorrhagic shock

Water sector disorders occur within well defined pathological situations. Losing large amounts of blood after multiple trauma leads to hypovolemia [15, 16]. Decreased extracellular compartment is partially compensated by transferring ionic liquids from the intracellular compartment (especially K^+^) [17]. The ultimate result of this state of self-compensation is global isotonic dehydration with hyperkalemia with a high risk of rapid hemodynamic decompensation. At the cellular level, tissue hypoperfusion causes anaerobic glycolysis producing lactate and reduced functional capacity of cells leading to death by necrosis or apoptosis [18]. Also, studies in laboratory animals have shown that the type of solutions used in resuscitation directly influence apoptosis in various tissues [19, 20]. One of the most significant side effects of hemorrhagic shock is represented by systemic inflammatory response syndrome (SIRS), appeared within hours post-trauma, being responsible for increased capillary permeability, release of proinflammatory factors, and release of highly reactive molecular species [19]. The levels of electrolytes with extremely high physiological importance are affected in hypovolemic shock. Altered electrolyte balance entails a multitude of cellular dysfunction with serious repercussions upon the patient in shock. After hemorrhagic shock, microcirculation shows significant dysfunction caused by capillary collapse, leading to decreased functionality and decreased tissue pO_2_ [20]. Nitric oxide (NO) is directly involved in pressure redistribution, a phenomenon explained by the ability to relax blood vessels [20]. For proper monitoring of patients with multiple trauma, studies recommend analyzing a series of parameters including temperature, skin perfusion, urine output, invasive blood pressure, heart rate, inflamatory markers and ABG parameters [21]. Following resuscitation fluids, even if macro-hemodynamic changes are favorable, the micro-hemodynamic changes can remain deficient. To monitor microvascular system, a parameter widely studied is the central venous oxygen saturation (ScvO_2_) [22]. ScvO_2_ is an important marker that shows the balance between oxygen delivery and oxygen consumption. However, ScvO_2_ monitoring may be misleading, particularly in regions with severe tissue hypoxia. To optimize fluid therapy based on microvascular system functionality, another parameter represented by central-venous-to-arterial CO_2_ difference was introduced (CO_2_ gap) [22, 23]. Physiologically, CO_2_ gap should be less than 5 mmHg. Studies have reported values above 5 mmHg in sepsis, severe hypovolemia and ischemia reperfusion syndrome. Elevated CO_2_ gap is associated with MODS and poor prognosis in critical patients [23, 24]. Usually tachycardia occurs due to vascular collapse and represents a compensatory physiological effects. Recently, it was shown that there may be special circumstances in this respect, when bradycardia occurs due to increased parasympathetic tone [25]. Lactic acidosis is a good indicator of tissue hypoxia even if vital signs are normal. Decreased lactate and lactic acidosis correction means restoring proper blood flow [26]. Base deficit represents one of the most important parameters in monitoring patients with multiple trauma and hemorrhagic shock. Base excess (BE) less than - 6 mmol/L indicates a possible intra-abdominal disease with massive blood loss or acute pulmonary failure [27]. Moreover there is a strong relationship between mortality and base excess in patients with multiple trauma and blood loss more than 50 % [28]. In addition, the following parameters are recommended to be monitored - mixed venous oxygen saturation, venous-arterial CO_2_ partial pressure, central venous pressure, pulmonary artery occlusion pressure, gastric tonometry or sublingual capnography [29].

### New ideas for volume replacement

The highest mortality rate in trauma patients is due to hemorrhagic shock and incorrect volume resuscitation [30]. Resuscitation is recommended to be guided by several clinical aspect, such as vital signs, urine output, perfusion, acid-base imbalance, immune function, extracellular matrix production and cell chemotaxis, ability to prevent cerebral edema, pulmonary edema [15]. The most important factor at the cellular level is the exchange pump Na^+^/H^+^ [31]. Wu et al., in a study conducted on a group of 24 pigs demonstrated that by blocking the pump Na^+^/H^+^ and thus reducing the level of lactic acid could protect cellular alteration [31]. Ischemia and reperfusion is an important precursor in the inflammatory processes that are responsible for producing oxygenated reducing agents. Saad et al., showed that the most affected in this regard are the lungs [32]. Impaired lung function is caused by deposits of fibrin, alveolar hemorrhage, interstitial fluid or neutrophil sequestration [32]. These combined lead to respiratory distress syndrome. Increased inflammatory response is stimulated by the presence of biochemical markers such as, interleukin 6 (IL-6), FR, which, biochemically has increased affinity to alveolar macrophages [7, 33]. Another important biochemical marker in this sense is tumor necrosis factor aflpha (TNF–alpha) which is produced 1–2 hours after hemorrhagic shock and is often associated with organ damage [34].

### Antioxidant therapy in volemic resuscitation

Numerous studies demonstrate that administration of antioxidants along with fluids leads to progressive increase in the rate of survival by significantly reducing oxidative stress and inflammatory response [35]. Numerous studies recommend administering substances with antioxidant capacity both in the first 24 hours post-trauma, as well as during the ICU admission. The most commonly used antioxidants are vitamin C, B, Selenium, N-acetylcysteine and resveratrol [14, 36]. Juretic et al., studied the implications of vitamins A, C and E on the inflammatory status. Following concomitant administration of 38 mg/kg Vitamin C (i.v.), 27 U/kg Vitamin E (i.v.) and 41 U/kg Vitamin A (i.v.), the group of animals who received antioxidant therapy showed significant reductions in inflammation and cardiovascular function potentiation [7]. Lloberas et al., studied the effects of administered vitamin C in laboratory animals with hemorrhagic shock. They reported improved kidney function and reduced systemic inflammation [37]. Another biologically active compound used as an adjunct to volume replacement solutions with outstanding results is Centhaquin. Ringer's lactate solution is effective in restoring hemodynamic parameters, but has a number of physiological limitations. Lavhale et al., have shown that the use of hypertonic saline solution with Centhaquin significant decreases the lactate concentration and support the increase and maintenance of mean arterial pressure [38]. He reports that the use of 0.05 mg/kg of Centhaquin is indicated for correct volume expansion [38]. The phenomenon is explainable by the pharmacological properties that it owns - central sympathetic activity and vasoconstriction, resulting in improved tissue reperfusion through cardiovascular control [38]. Numerous studies recommend glutamine as antioxidant in critical patients. However, there are a series of side effects after high doses of glutamine. Daren et. al performed a study in 40 intensive care units from Europe and North America and emphasized a direct correlation between glutamine administration and MODS incidence. [39].

Aksu et al., explained the benefits of volume resuscitation when using gluconate - balanced crystalloid which prevent hyperchloraemic acidosis, anion gap and renal hypoperfusion [40]. They highlighted the decline in tissue inflammation but also the increase of favorable parameters for volume and electrolyte balance [40]. Another intense studied compound is decay accelerating factor (CD55/DAF) [30]. It is a membrane protein that has enzymatic action , protecting the cells of antologous complement activation. Lucca et al., conducted a study in which hemorrhagic shock resuscitation was performed using recombinant human DAF. Histological analysis demonstrated that the use of DAF (50 mg/kg) in volume replacement solutions reduce particulary renal and hepatic injuries. In addition to these benefits, the use of DAF reduces the need for fluids and prevents inflammatory responses and coagulopathy [30]. A biologically active compound used in combination with fluids is valproic acid [41]. Hemorrhagic shock blocks histone acetyltransferase activity, suppressing gene transcription. Valproic acid acts upon histone deacetylase inhibitor [41, 42]. Glycogen Synthase Kinase (GSK) [43, 44] is a biologically active substance used in the management of fluid resuscitation. This enzyme regulates a number of cellular functions - protein synthesis, cell differentiation and cell motility. Overexpression of GSK lead to an extremely complex inflammatory phenomenon [43]. Recent studies show that by blocking the activity of GSK-3-Beta, inflammation and hepatocellular injury markers decrease significantly. To inhibit the activity of this enzyme, Jellestad et al., used two agents: 4-benzyl-2-methyl-1,2,4-thiadizolidine 3,5-diones (TDZD-8) [34] and domethyl sulfoxide (DMSO). In the study they demonstrated that inhibition of GSK−3−Beta enzyme leads to increased liver function and microcirculation [43].

### Fluid resuscitation and inflammatory and redox status

Fluid management in trauma patients is extensively studied, and controversies regarding side effects, inflammatory response and tissue hypoxia further complicate the clinical decisions. Table 1 presents studies related to fluid management in critical patients.

**Table 1:** Fluid resuscitation studies

Author	Type of fluid	Observations	Reference
O’Malley et al.	NaCl 0.9 %, respective lactated Ringer	They noticed that when using lactated Ringer solution, the incidence of patients with hyperkalemia decreases, as well as incidence of acidosis.	[65]
Maitland et al.	Albumine, respective NaCl 0.9 %	In both cases there was an increase in mortality rate.	[66]
Shaw et al.	NaCl 0.9 %, respective balanced crystalloid	When using NaCl 0.9 % there was an increase in mortality rate and ABG imbalances.	[67]
Annane et al.	Comparison between colloids and cristaloides.	There was no statistically significant difference between the two groups.	[68]
Rasmussen et al.	Studied the effects of HES 130/0.4 upon coagulation	They noticed reduced clot strength. It also showed a decrease in fluid requirements.	[69]
Abeed et al.	Studied the renal function after NaCl 0.9 % vs. Plasma-Lyte 148	There was a decrease renal blood flow and renal cortical tissue perfusion in case of NaCl 0.9 % administration.	[70]

German Society for Trauma Surgery recommends using Ringer's solution acetated or malate Ringer's solution before the start of volume resuscitation. The metabolism of acetate prevents hyperchloraemic acidosis, being metabolized to carbon dioxide and water. Meanwhile, acetate molecule can be metabolized in gluconeogenesis, forming bicarbonate ion (HCO_3_^−^). For each functional group in acetate molecule (COOH), one HCO_3_^−^ molecule is produced. Biochemical reaction occurs mainly in muscle and liver. Röhring et al., have examined this recommendation, demonstrating that after induction of hemorrhagic shock in laboratory mice, the use of acetated Ringer's solution achieved a successful resuscitation level [45]. During shock, arterial pH was in average around 7.2. Administration of acetated Ringer's solution, unlike saline, resulted in significantly higher pH. The TNF-alpha and IL-6 had lower values [45].

Recent international guidelines and protocols suggest administration of pressor substances in order to ensure tissue perfusion. Such an agent is arginine vasopressin, with antidiuretic and suppressor of NO synthesis. A lot of studies recommend using vasopressin due to reduced side effects, but also because it brings beneficial effects in fluid management [46].

A negative factor post-resuscitation is the volume of fluid administered intravenously per unit time [47]. Yu et al., have studied post-resuscitation differences after performing slow resuscitation (12 hours) and rapid resuscitation (30 minutes) [48]. Slow and continuous resuscitation decrease proinflammatory cytokine, microvascular damage and hypoalbuminemia. At the same time pulmonary disorders are less often encountered [48].

A major problem during fluid resuscitation is ensuring the functionality of microvascular system. Impaired capillary system leads to tissue hypoxia, responsable of inflammation, mitochondrial dysfunctions, increased FR and cellucar apoptosis. Dubin et al. studied the effects of different types of fluids upon microvascular system in septic patients. The fluids used in the study were HES 130/0.4 and NaCl 0.9 %. They reported an increased capillary microvenous flow index and perfused capillary density in patients who received HES 130/0.4 [49]. Wang et al., demonstrated that the use of whole blood instead of saline solutions reduce the inflammatory response of the lung tissue [50].

One aspect leading to complicated post-resuscitation evolution and also to lower survival rate is delayed resuscitation [51]. Chien et al., studied the effects occurring by delaying resuscitation. Thus, they followed the activation profile of cytokines according to the delay time in laboratory animals suffering from hemorrhagic shock. After analyzing the results, they reported increased levels of IL-6 if resuscitation started after 45–60 minutes after hemorrhagic shock [52].

An important trauma that causes haemorrhagic shock is traumatic brain injury [53]. Studies have shown that in the case of severe brain injury, the main cause of death is massive haemorrhage [54, 55]. Standard treatment for volume replacement is rapid administration of the crystalloid solutions. It is demonstrated that this method of resuscitation with saline isotons, affects patient due to ischemic brain injury and haemodilution [56].

A pharmaceutical compound found in broad based research is haemoglobin oxygen carriers (HBOCs) described in the literature as the most ideal restoring tissue perfusion [57]. Such a complex is HBOC-201-polymerised bovine HBOC [58]. Curry et al., simulated in the laboratory a 30 minutes pre-hospital time. During this time, the complex HBOC-201 is administered for resuscitation. Histological and laboratory tests have shown that the infusion of this complex increase cerebral oxygenation and perfusion while renal failure and acute lung injury were minimized [58].

There are a number of controversies and contradictory opinions regarding the management of patients with burns. In patients with major burns, volume replacement involves extra attention due to increased electrolyte imbalances and biochemical/biophysical dysfunctions [59] – hypovolemic organ failure, incidence of shock, acute renal disease, lung failure by altering the ventilation/perfusion ratio, digestive tract dysfunction by bacterial translocation or erythrocyte hemolysis by membrane hyperoxidation [60]. There is also a generalized alteration of cell membranes. Fluid administration should be adjusted according to each patient depending on the biological and physiological results [61]. Arlati et al., reported that patients with major burns who received Parkland formula developed multiple organ dysfunction more quickly as opposed to permissive hypovolemia and mean arterial pressure of 70 mmHg [62]. A variety of other studies reveal that the Parkland method produces a series of complications – acute respiratory distress or abdominal compartment syndrome, and is associated with death in up to 70 % of cases. Hypertonic solutions commonly used in such cases contain 240–300 mEq/L Na^+^) resulting in hypernatremia and hyperosmolarity [60].

All these dysfunctions lead to renal dysfunction, intestinal oxidative stress and increased vascular permeability. Increased vascular permeability is a major cause of fluid loss and increased osmotic pressure in the burned tissue, inducing edema and hypoproteinemia. Sun et al, conducted a study on the use of hypertonic saline solution (200 mEg/L Na^+^) for resuscitation in the laboratory mice with multiple burns. The analyses reported a significant decrease in pulmonary edema incidence. Meanwhile biosynthesis of inflammatory mediators significantly decreases. By lowering the concentration of cytokines, the liver injury is reduced, thus inhibiting cyclooxygenase. The use of such hypertonic solutions (200 mEg/L Na^+^), corrects hyponatremia induced by severe burns [60]. Hypoproteinemia accentuate edema, therefore albumin supplementation is required. The altered coagulation disorders are rebalanced by infusion of fresh frozen plasma. Administration of whole blood is not required due to increased viscosity.

Guidry et al., have studied the differences between the colloid and crystalloid solutions [63]. They recommend lower volumes of colloid and increased volumes of crystalloid to reduce mortality and increase survival rate [63].

Obstetric patients are a real challenge in the event of polytrauma with hemorrhagic shock [64]. Yu et al., conducted a study on pregnant experimental models. The basic idea of the study refers to volume replacement to maintain mean arterial pressure above 60 mmHg [64]. The biological tests performed did not find significant correlations between blood transfusion and prothrombin time, activated partial thromboplastin time and fibrinogen. When using Ringer’s solution, significant and positive correlations were revealed regarding prothrombin time and activated partial thromboplastin time and no correlations for fibrinogen [64].

## Conclusions

Hemorrhagic shock has been shown to produce a series of inflammatory reactions affecting cells, biochemical and physiological metabolism and ultimately organ functions. Septic shock, a phenomenon common in most trauma patient, produces severe decompensation in terms of hemodynamics due to low vascular resistance, myocardial dysfunction, oxidative stress, overproduction of NO, microcirculation disorders or excessive activation of K^+^-ATP sensitive channel. The literature does not offer exact guidelines for fluid resuscitation, and there is a lot of controversy regarding the pros and cons of using colloids or cristalloids. In conclusion, we can say that for each patient there is a resuscitation management to be chosen according to clinical and traumatic status, as well as to the biological and physiological investigations.

## Abbreviations

pO_2_: partial pressure of oxygen
NO: nitric oxide
ABG: arterial blood gases
BE: base excess
CO_2_: carbon dioxide
TNF–alpha: tumor necrosis factor alpha
CD55 / DAF: decay accelerating factor
COOH: acetate molecule
HES 130/0.4: hydroxyethyl starch
HCO_3_-: bicarbonate
IL–6: interleukin 6
GSK: glycogen synthase kinase
TDZD 8: 4-benzyl-2-methyl-1,2,4-thiadizolidine 3,5-diones
DMSO: domethyl sulfoxide
HBOCs: haemoglobin oxygen carriers
ICU: Intensive Care Unit
FR: free radicals
OS: oxidative stress
